# Impact of COVID-19 on the Use of Emergency Contraceptives in Ghana: An Interrupted Time Series Analysis

**DOI:** 10.3389/frph.2022.811429

**Published:** 2022-02-04

**Authors:** Kamil Fuseini, Leah Jarvis, Michelle J. Hindin, Kofi Issah, Augustine Ankomah

**Affiliations:** ^1^Population Council, Accra, Ghana; ^2^Population Council, New York, NY, United States; ^3^Family Health Division, Ghana Health Service, Accra, Ghana

**Keywords:** COVID-19, family planning, emergency contraception, oral, Ghana

## Abstract

The Coronavirus disease pandemic has disrupted reproductive health services including decline in the use of pre-coital contraceptives. However, evidence of its impact on the use of emergency contraceptives, often, post-coital methods, is limited in the emerging literature, hence this study. Data on total number of emergency contraceptive users from January 2018 to February 2020 (pre-pandemic) and March to December 2020 (during the pandemic) were extracted from the Ghana Health Service District Health Information Management System. Interrupted Time Series analysis was used to estimate the impact of the pandemic on the trend of emergency contraceptive use, adjusting for serial autocorrelation and seasonality. The results showed a gradual upward trend in emergency contraceptive use before the pandemic, increasing at a rate of about 67 (95% CI 37.6–96.8; *p* = 0.001) users per month. However, the pandemic caused a sudden spike in the use of emergency contraceptives. The pandemic and its related restrictions had an immediate effect on the use of emergency contraceptives, increasing significantly by about 1939 users (95% CI 1096.6–2781.2; *p* = 0.001) in March 2020. Following March 2020, the number of emergency contraceptive users continued to increase by about 385 users per month (95% CI 272.9–496.4; *p* = 0.001). The evidence shows that use of emergency contraceptives, often used as post-coital methods for unprotected sex was not negatively impacted by the pandemic. In fact, it is the opposite. Hence, in planning for similar situations attention should be given to the distribution of post-coital contraceptive methods.

## Introduction

Infectious disease outbreaks often disrupt economic, social, educational, and health systems (1–3).

In March 2020, the World Health Organization declared the coronavirus disease of 2019 (COVID-19) a global pandemic. Worldwide, the burden of the pandemic on health systems has impacted Sexual and Reproductive Health (SRH) services and programs among others (4).

SRH services have been affected by governments' response to the pandemic, such as lockdowns, stay at home and/or work from home directives, physical distancing, and travel restrictions (4–6). Even where there are only limited COVID-19 related restrictions, the fear of contracting the virus may limit client's use of health facilities for non-essential services such as contraceptives (7, 8).

Studies have shown that infectious disease outbreaks have negative implications for the uptake of contraceptive services. For instance, at the peak of the Ebola epidemic, distribution of contraceptives declined significantly in Liberia by 65% and in Sierra Leone by 23% (1). Specific contraceptive methods such as implants, male condom, pills, and injectables showed similar declines (1). A study in rural Guinea also showed that utilization of contraceptive services decreased by half, from a monthly average of 531 visits before the Ebola epidemic to 242 visits in the peak month of the Ebola outbreak (9). Similar dips in FP visits were observed across the different FP methods (9).

Specific to the COVID-19 pandemic, studies have shown its negative impact on the uptake of various contraceptives (10, 11). It has been estimated that the potential impact of COVID-19 on contraceptive use could result in a fall of about 60 million fewer users of modern contraception worldwide in 2020 (11). In Mozambique, the imposition of the state of emergency due to COVID-19 led to a modest short-term drop in both FP service provision and utilization, although it was followed by a relatively rapid rebound (12). In Bangladesh, COVID-19 had a sustained disruption of FP service provision in the short- and mid-term analyses of trends before, during, and after COVID-19 lockdowns (13). Specifically, long-acting reversible contraceptives (LARCs) were more severely affected, with severe immediate impacts on implant and IUD service provision with shorter-acting methods declining at a smaller magnitude (13).

Indeed, the impact of the pandemic on contraceptive use depends on several factors such as the methods used by couples, types of disruptions experienced, and their need for contraceptives at a particular point in time (11). However, the literature has almost exclusively focused on the impact of COVID-19 on the use of pre-coital contraceptive methods, with limited attention given to the impact of COVID-19 on the use of post-coital contraceptives (2, 10–13). Hence, there is limited empirical evidence on the impact of infectious disease outbreaks on the use of emergency contraceptives (ECs) in a pandemic situation.

Ghana confirmed its first two cases of COVID-19 on March 12, 2020. As a result, the government, and stakeholders started the implementation of the Ghana Emergency Preparedness and Response Plan (2020). Under the Emergency Powers Act and the Public Health Act (Act 851), the government restricted mass religious, social, and sporting gatherings suspended international travel, closed educational institutions at all levels, and imposed a partial lockdown on the metropolitan localities of Accra, Kumasi, Tema, and the environs of Kasoa. The partial lockdown lasted for 3 weeks and was lifted on 19^th^ April 2020. However, the other restrictive and preventive protocols continued (14).

ECs are very important in the contraceptive method mix. It is often used by women who have had unprotected sex or as a backup in cases of perceived failure of traditional methods like withdrawal or condom breakage (15–19). In some African countries, ECs are provided in both public and private health facilities; however, they are mostly accessed through private pharmacies and chemical drug stores (20), usually due to convenience, quickness, and confidentiality (21).

In Ghana, national-level surveys have shown that knowledge of ECs among all women increased from 28.2% in 2003 (22) to 68.7% in 2017 (23). In 2017, the current use of ECs was 1.3% among all women, 0.9% among currently married women, and 5.4% among sexually active unmarried women (24). Small-scale studies in Ghana have also shown similar results. In a study to assess the awareness, use, and associated factors of ECs among women of reproductive age in northern Ghana, 63% of students in a university had heard of ECs and 37% had used it (25). Another study found that among female senior high school students in Ho municipality in the Volta Region of Ghana, knowledge of ECs was 98.8%, while 57.1% of students had a history of EC use (26). Furthermore, a study among young people aged 15–24 years in Accra, found that ECs were the second most popular modern contraceptive method among women, next to condoms (27). Another study in Accra revealed that of all sexually experienced women in the study, 14.5% reported having ever used EC at any point in time (16).

ECs are preferred as convenient post-coital methods (18, 28), and while evidence from elsewhere shows that infectious disease outbreaks including COVID-19 impact negatively on the use of specific contraceptive methods, there is limited empirical evidence on the impact of COVID-19 on the use of ECs specifically. This study sought to assess the impact of the Covid-19 pandemic on the use of ECs, in a country where knowledge of EC is high (24, 27, 29) by comparing pre pandemic data with data during the early phase of the pandemic. Given that in Ghana, sexual intercourse is often unplanned (18), and COVID-19 risk mitigation strategies led to people staying at home, this may lead to an increase in EC use, as it is widely available inside and outside of health facilities within communities.

## Materials and Methods

### Study Design

The study used the Interrupted Time Series (ITS) design. This design can be used to make comparisons across time within a population. Generally, it is applied to natural experiments with an intervention introduced at a known point in time (30). The ITS study design uses data collected at regular intervals over time, and can be used for a pre-post comparison while accounting for underlying trends in the outcome variable (30, 31).

### Data

This study utilized data on EC users extracted from the Ghana District Health Information Management System (DHIMS2) database in February 2021. DHIMS2 is a web-based application for remotely compiling data across different levels of the health system (i.e., public, and private including pharmacies and chemical stores) to a central storage point. The system is used to capture data on various health service indicators including the different types of contraceptive methods (e.g., implants, condoms, IUD, injectables, and ECs) (32). The extracted data were on EC users from January 2018 to December 2020. See details of the distribution of EC users over the study period in Table 1. The data were exported to STATA version 16 for analysis.

**Table 1:** Distribution of EC users by month in Ghana, 2018–2020.

**Month**	**2018**		**2019**		**2020**	
	**%**	***n***	***%***	***n***	***%***	***n***
January	4.5	692	4.4	1,059	2.4	1,658
February	4.0	618	4.7	1,124	4.5	3,104
March	9.1	1,421	7.9	1,920	6.1	4,199
April	10.9	1,693	9.0	2,178	7.4	5,099
May	10.9	1,697	7.1	1,716	8.9	6,094
June	15.0	2,337	9.1	2,209	8.4	5,773
July	13.2	2,047	10.8	2,600	10.2	6,957
August	9.7	1,505	8.4	2,018	12.5	8,578
September	5.5	854	7.9	1,912	8.3	5,684
October	5.8	897	11.1	2,676	10.2	6,977
November	6.8	1,057	9.1	2,188	10.1	6,950
December	4.8	742	10.6	2,567	10.9	7,446
**Total**	**100.0**	**15,560**	**100.0**	**24,167**	**100.0**	**68,519**
**Mean**		**1296.7**		**2013.9**		**5709.9**

*Source of data, DHIMS2, Ghana*.

### Variables

The outcome variable of interest was use of EC, defined as the total number of FP clients who used ECs per month over the period under study. Use of EC was measured as a continuous variable. Months were included in the analysis to control for seasonality measured as dummy variables (1 = Yes, 0 = otherwise), using January as the reference category.

### Statistical Analysis

To assess the impact of the pandemic on the use of ECs, trends in the monthly number of EC users were assessed before and during the COVID-19 pandemic. The study period was divided into two phases: 26 months of pre-COVID-19 data (January 2018 to Feb 2020) and 10 months of data during COVID-19 (March 2020 to December 2020), which is enough to have sufficient power (34). With the onset month of COVID-19 and its preventive measures (March 2020) as the start of the event, ITS segmented ordinary least square regression model was estimated to assess the impact of COVID-19 on the use of ECs. The analysis was conducted using the Prais–Winsten method to account for the effect of serial autocorrelation (33) and took into consideration the potential seasonal effect of ECs uptake by including months as dummy indicator variable in the model. The interrupted time series regression model (single group) takes the form of:


$$\begin{array}{l} Y_{t}=\beta_{0}\;+\beta_{1}T_{t}\;+\beta_{2}X_{t}\;+\beta_{3}X_{t}T_{t}\;+\;\epsilon_{t} \end{array}$$

Where Y_t_ is the aggregated outcome variable measured at each equally spaced time point t. β_0_ represents the intercept or starting level of the outcome variable (estimated number of EC users at the beginning of the pre-outbreak period). β_1_ estimates the monthly change in the number of EC users until the onset of the COVID-19 pandemic. T_t_ is the time since the start of the study. β_2_ represents the change in the level of EC use that occurred in the period immediately following the onset of COVID-19 (compared with the counterfactual). β_3_ represents the difference between the trend in EC use pre-COVID-19 and during COVID-19 periods. X_t_ is a dummy (indicator) variable representing the onset of COVID-19 (pre-COVID-19 period 0, otherwise 1). X_t_T_t_ is an interaction term and ϵ_t_ is the random error term. The ITSA analysis was implemented using the “itsa” command in STATA (33).

## Results

Table 1 shows the distribution of EC users by month from January 2018 to December 2020. In general, EC use increased across the 3 years. Use of ECs from January 2018 to December 2020 was 108246 (15560 in 2018, 24167 in 2019, and 68519 in 2020). The number of EC use increased from 1296.7 in 2018 to 2013.9 in 2019 and further increased to 5709.9 in 2020. In 2018, the highest proportion of EC use was in June (15%), followed by July (13.2%), and April (10.9%) and May (10.9%). The lowest proportion of EC use was in February (4.0%). In 2019, use of EC was highest in October (11.1%) and lowest in January (4.4%). In 2020, use of EC was highest in August (12.5%) and lowest in January (2.4%).

**Figure 1:**
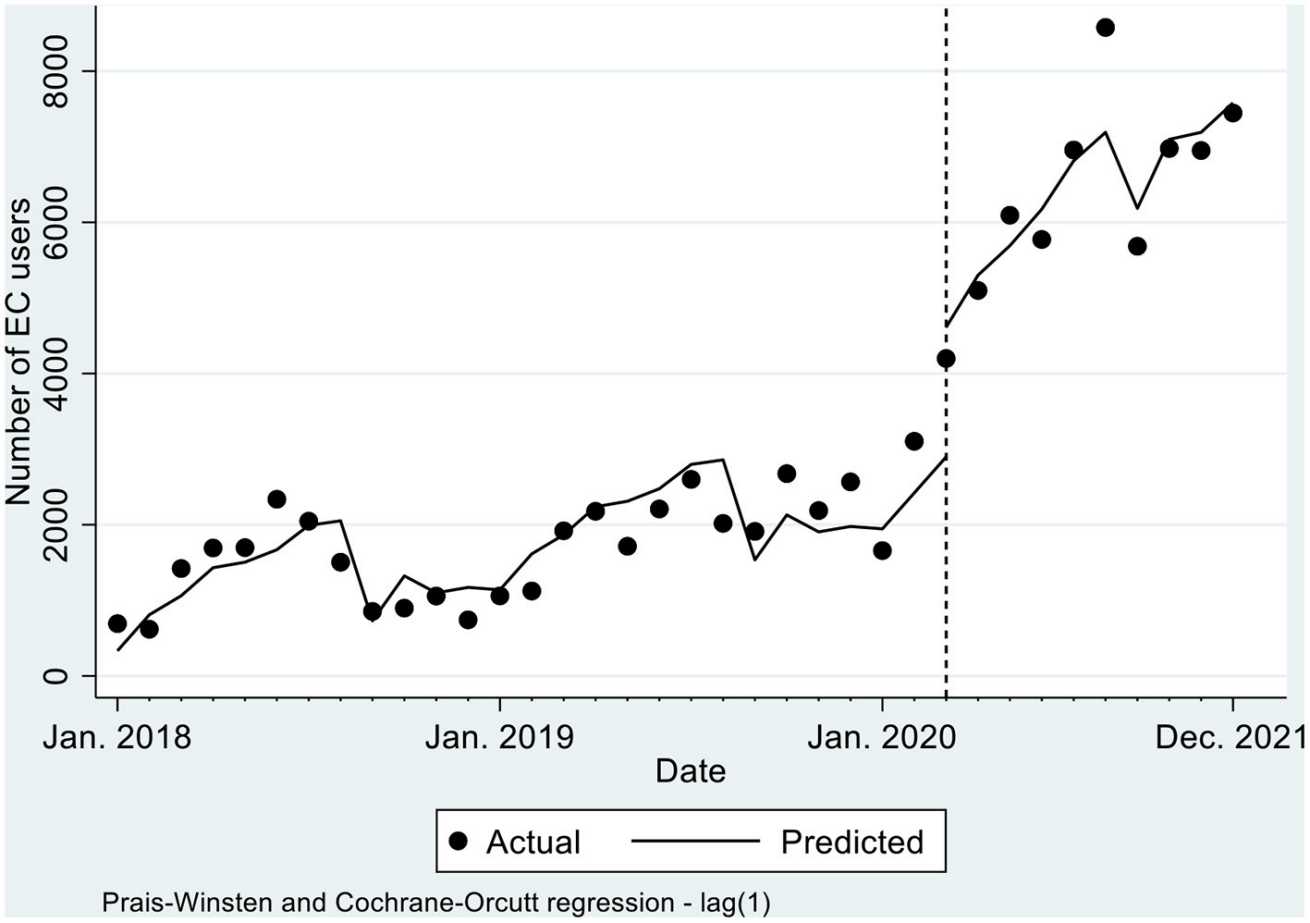
Actual and predicted trends in total number of EC users, by month, 2018-2020.

Table 2 visualized in Figure 1 shows EC use over the 26 months (January 2018 to Feb 2020) before the first COVID-19 confirmed case in Ghana and 10 months of data since the first confirmed case of COVID-19 (March 2020 to December 2020). It was estimated that in January 2018 (at baseline), there were 333.2 EC users [95% CI (-240.2, 906.5)]. I In each month, from February 2018 to March 2020, the number of EC users rose significantly by 67 users per month [p ≤ 0.001, 95% CI (37.6–96.8)].

**Table 2:** Parameter estimates of the impact of COVID-19 pandemic on EC users in Ghana, 2018–2020.

	**Coef**.	**S. E**.	**95% C. I**.	
Number of EC users in Jan. 2018	333.2	275.7	−240.2	906.5
Monthly change in number of EC users, Jan. 2018-Feb. 2020	67.2^***^	14.2	37.6	96.8
Change in the level of EC users in Mar. 2020	1938.9^***^	405.0	1096.6	2781.2
Change in trend in monthly number of EC users between Jan 2019-Feb. 2020 compared to Mar.-Dec. 2020	317.4^***^	53.4	206.3	428.6
Monthly change in number of EC users from Mar.-Dec. 2020	384.6^***^	53.7	272.9	496.4
Month				
January (Reference category)				
February	408.4	418.9	−462.7	1279.5
March	593.1	302.6	−36.2	1222.5
April	896.8^**^	276.7	321.3	1472.3
May	902.8^*^	401.3	68.3	1737.2
June	1000.5^*^	429.5	107.3	1893.6
July	1255.8^***^	245.3	745.7	1765.9
August	1248.5	744.7	−300.3	2797.2
September	−141.5	314.8	−796.2	513.1
October	385.4	402.9	−452.4	1223.3
November	94.7	282.8	−493.5	682.9
December	99.2	455.5	−848.1	1046.6
rho	−0.07146			
Durbin-Watson statistic (original)	2.10913			
Durbin-Watson statistic (transformed)	1.97073			

* *p < 0.05*;

** *p < 0.01*;

*** *p < 0.001*.

*Source of data, DHIMS2, Ghana*.

When the COVID-related restrictions began in March 2020, there was a significant increase in the number of EC users by 1938.9 [*p* ≤ 0.001, 95% CI (1096.6, 2781.2)] compared to the pre COVID-19 level. Comparing the pre-COVID-19 trend to post-COVID-19; there was a statistically significant increase in the monthly trend of EC users by 317.4 per month [*p* ≤ 0.001, 95% CI (206.3, 428.6)]. Similarly, after March 2020, there was a significant monthly increase in EC users at a rate of 384.6 per month [*p* ≤ 0.001, 95% CI (272.9, 496.4)]. Efforts were made to conduct further sub-national analysis by Ghana's 16 regions, however, there were not enough data in some regions to conduct robust analyses.

## Discussion

To the best of our knowledge, this is the first study to use routine service data to examine the impact of infectious disease outbreaks on ECs. This study used routine service data from DHIMS to assess the impact of the COVID-19 pandemic on the use of ECs in Ghana. The study considered January 2018 to February 2020 as the pre-COVID-19 period, and March to December 2020 as the COVID-19 period, and March 2020 as the onset month of the start of the event. Studies assessing the impact of infectious disease outbreaks on contraception have often considered pre-coital contraceptives. These studies have found that infectious disease outbreaks (including COVID-19) impact negatively on the use of contraceptives (i.e., oral contraceptives, injectables, IUD, and implants) (1, 9). However, the results of this study revealed that the COVID-19 pandemic and its associated preventive measures rather led to an increase in the use of ECs in Ghana.

Although the trend in EC use before the COVID-19 pandemic was increasing significantly by about 67 users per month, in March 2020 (the interruption month), the number of EC users increased significantly to levels never observed before the pandemic (increased by 1,939 additional users). During the COVID-19 period, the number of EC users was increasing by about 385 users per month, resulting in a difference in trend between pre-and post-COVID-19 of about 317 EC users monthly. This is an indication that COVID-19 may have led to a sustained increase in the use of ECs in Ghana.

Various factors may explain these findings. The COVID-19 risk mitigation strategies starting from March 2020, emphasized on staying at home; hence, partners would be exposed to an increase in sexual activity. Evidence show that crisis or instability usually leads to an increase in unplanned pregnancies. For example, in 2006, the Yogyakarta earthquake in Indonesia resulted in the decrease in use of contraceptives leading to an increase in unplanned pregnancies (35). Hence, the inability of Ghanaian couples to plan for sexual encounters may have led to unprotected sexual encounters (18). To prevent unintended pregnancies, ECs may have become a convenient option as post-coital contraception with minimal side effects (15, 18, 21). The restriction in movements making partners stay at home may have increased unplanned and unprotected sexual intercourse resulting in the increase in use of ECs. Put together, these factors may explain the sustained increase in ECs use between March and December 2020. It is important to note that public and private health facilities provided health services including contraceptives during the COVID-19 pandemic period. ECs were readily available since restrictions in movement did not include visits to government and private pharmacies to procure products.

The findings of this study show that the impact of infectious disease outbreaks on use of EC may differ from what other studies have found about other methods of contraception, specifically pre-coital methods. While studies show that pandemics have a negative impact on use of contraceptives such as implants and IUDs, this study shows that the COVID-19 pandemic may have led to an increase in use of ECs. The data used for this analysis is routine service data; hence, there may have been disruptions in data capturing during the COVID-19 period, thus the increase in EC use could have been underestimated.

## Limitations

Repeat use of ECs could have led to double counting, however, evidence in Ghana shows that multiple use of ECs within a month is minimal (16). There was also limited data in DHIMS on socio-demographic characteristics, where available, it did not lend itself to these kinds of analyses. This limitation notwithstanding, the statistical analyses are appropriate and robust and allow for interpretation of results to inform decision-making.

## Conclusion

The findings of this study demonstrate that the COVID-19 pandemic has had an impact resulting in the increase in use of ECs in Ghana. Studies show that use of pre-coital contraceptives has been found to decline during epidemics and pandemics. However, the findings of this study demonstrated the opposite for EC use, hence, policymakers, and service providers should prioritize the provision of ECs in similar situations. With evidence form other studies showing that access and utilization of other forms of contraceptives being impacted negatively, and scarce resources being channeled to COVID-19 response, the contribution of ECs to the overall contraceptive uptake in Ghana should not be neglected.

## Data Availability Statement

Publicly available datasets were analyzed in this study. This data can be found here: The data used for this study are available from Ghana Health Service, upon reasonable request.

## Ethics Statement

The project received ethics approval from the Ghana Health Ethics Review Committee (GHS-ERC 007/03/20).

## Author Contributions

KF, LJ, MH, KI, and AA were involved in the conceptualization of the manuscript and methods, involved in the write-up, and review of the final manuscript. KF conducted the data analysis. All authors contributed to the article and approved the submitted version.

## Funding

This work was supported by the Bill and Melinda Gates Foundation (ID: INV-000782).

## Conflict of Interest

The authors declare that the research was conducted in the absence of any commercial or financial relationships that could be construed as a potential conflict of interest.

## Publisher's Note

All claims expressed in this article are solely those of the authors and do not necessarily represent those of their affiliated organizations, or those of the publisher, the editors and the reviewers. Any product that may be evaluated in this article, or claim that may be made by its manufacturer, is not guaranteed or endorsed by the publisher.
